# Trauma patients ≥ 65 years old constitute 40% of severely injured adult trauma patients in Sweden – a 10-year analysis of geriatric trauma prevalence

**DOI:** 10.1007/s00068-026-03226-0

**Published:** 2026-06-01

**Authors:** Sara Bredberg, Emelie Modalen, Charlie Jöneby, Denise Nilsson, Oscar Lapidus

**Affiliations:** Department of Clinical Science, Intervention and Technology, Karolinska Institutet, Stockholm, Sweden

**Keywords:** Trauma, Geriatric, Temporal, Trend, Age, Injury, Swedish Trauma Registry

## Abstract

**Background:**

The elderly population in Sweden is steadily increasing. Age is an independent predictor of mortality in trauma patients. There is growing evidence that geriatric patients constitute an increasing proportion of the Swedish trauma population. No studies have analyzed temporal trends in the proportion of geriatric trauma patients in the context of the national trauma population in Sweden.

**Aim:**

To determine the proportion of geriatric patients in the Swedish trauma population and analyze temporal trends in geriatric trauma prevalence.

**Methods:**

The study cohort was 11,969 severely injured trauma patients (NISS > 15) treated in Sweden during 2013–2022, obtained from the Swedish Trauma Registry. The proportion of geriatric patients was compiled annually. Temporal trends were analyzed using weighted linear regression models. Two subgroups were defined based on injury severity (NISS ≥ 25 and NISS ≥ 40).

**Results:**

The proportion of patients ≥ 65 years in the adult trauma population increased from 30% to 40% during 2013–2022 (*p* = 0.003), while the proportion of patient ≥ 80 years also increased, from 8.6% to 17% (*p* = 0.009). Similar increases were also seen in the NISS ≥ 25 and NISS ≥ 40 subgroups.

**Conclusion:**

There is likely an increase in geriatric trauma prevalence in Sweden, but the full extent of this temporal trend remains uncertain. Trauma patients ≥ 65 years old constitute 40% of severely injured adult trauma patients in Sweden. Further investigations of geriatric trauma prevalence in relation to demographic changes may be of interest to determine the future implications for Swedish healthcare.

## Background

The Swedish population is steadily aging, with the most significant growth among individuals aged 80 years and older [1]. By 2030, the proportion of the Swedish population ≥ 65 years old is projected to have reached approximately 22% [2]. Meanwhile, activity levels and independence among older individuals are higher than in previous decades, putting them at risk of sustaining traumatic injuries [3]. Elderly patients also disproportionally contribute to the burden of injury-related costs compared to their younger counterparts, accounting for a substantially higher proportion of total healthcare expenditures [4].

Age is an independent predictor of death in trauma [5–7]. In in combination with a high abundance of comorbidities in the elderly population, this may introduce significant challenges to trauma care, as elderly patients constitute an increasing proportion of the severely injured trauma cohort in developed countries [8–12]. Trauma has traditionally been considered to be a disease of the young, but 42% of traumatic deaths in adults in Sweden between 1999 and 2012 occurred in people aged 65 years or older, highlighting the clinical burden of geriatric trauma [13].

In Sweden, few studies have examined the effects of this demographic shift on the general trauma population, but some authors suggest that geriatric patients may constitute an increasing proportion of the Swedish trauma population [14, 15]. One recent study found that 15% of severely injured patients at the regional trauma center in Stockholm, Sweden had injuries sustained from low-energy mechanisms, albeit without any temporal trends during the study period [15]. However, no studies have analyzed temporal trends in the proportion of geriatric trauma patients in the context of the national trauma population in Sweden.

## Aim

To determine the proportion of geriatric patients in the Swedish trauma population and analyze temporal trends in geriatric trauma prevalence.

## Methods

### Study design

This was a retrospective registry-based study, reported in accordance with the STROBE guidelines [16].

### Ethical considerations

The study was approved by the Ethical Review Authority in Sweden (2024-02482-01, 2022-06727-01).

### Setting

The present study examined trauma patients treated in Sweden, a developed northern European country with a population of 10.5 million people in 2022 [1]. The Swedish Human Development Index (HDI) is 0.959, ranking fifth globally [17]. In 2022, 88% of inhabitants resided in urban areas, and the proportion of people ≥ 65 years and ≥ 80 years (out of everyone ≥ 15 years old) was 25% and 6.7% [1]. The overall proportion of people ≥ 65 and ≥ 80 years old was 20% and 5.5% [1].

The Swedish healthcare system is publicly funded through the taxpayers and organized in a decentralized model, with each of the 21 regions responsible for healthcare for their inhabitants; there are also six larger healthcare regions which are geographic subdivisions which coordinate healthcare between regions [18]. Sweden has no uniform designation of trauma centers nationally but each of the larger healthcare regions have varying degrees of prehospital selection of trauma to their respective university hospitals, where specialized care is centralized [19].

### Participants

The study cohort included all trauma patients ≥ 15 years old treated in Sweden during 2013–2022 with a New Injury Severity Score (NISS) > 15. For patients who were secondarily transferred between hospitals, only the initial registration from the first hospital was included to remove duplicate registrations from analyses. Subgroup analyses of the most severely injured patients were also performed using NISS ≥ 25 and NISS ≥ 40 as threshold values to define these populations.

### Variables

The primary outcome was the proportion of geriatric trauma patients each year, which was calculated by dividing the number of trauma patients ≥ 65 years old by the total number of patients in the severely injured trauma cohort annually. The secondary outcome was the change in proportion of patients ≥ 80 years old. Age was obtained from SweTrau using the ‘pt_age_yrs’ variable in the registry. Injury severity was defined using the ‘NISS’ variable obtained from the registry. Secondary transports were defined as patients with either ‘pre_transport’=9999, ‘TraumaAlarmCriteria’=9999 or ‘host_transfered’=2 and excluded from analysis.

### Data sources

This study analyzed data from the Swedish Trauma Registry (SweTrau), a national quality registry which collects and distributes data with the aim to monitor and improve trauma care in Sweden [20]. Patients are included in the registry if they prompt an in-hospital trauma team activation (TTA) on arrival to hospital or if they are found to have NISS > 15 regardless of TTA status [21]. As of 2022, all 49 emergency hospitals in Sweden were connected to SweTrau, and in 2023 the registry was reported to have a national coverage of approximately 85% [22].

### Bias

The present study included patients treated hospitals in all of Sweden; however, the national coverage of the SweTrau registry increased during the study period. This study is subject to time-varying ascertainment bias, where temporal trends in geriatric trauma prevalence may be the result of increasing registry coverage and inclusion of hospitals with a higher proportion of geriatric trauma patients. However, as SweTrau coverage has only increased over time, the present study still provides insights regarding the demographics of the Swedish trauma cohort. This is also further elaborated on in the Limitations section of the manuscript.

### Study size

All patients registered in SweTrau during the study period were assessed for eligibility based on the study inclusion and exclusion criteria. Including patients prior to 2013 was deemed unfeasible due to uncertainty regarding data quality.

### Statistics

Linear regression weighted to the number of cases each year was then used to evaluate temporal trends in geriatric trauma prevalence during the study period, as well as to assess temporal trends in the median age of the trauma population. The weighting was performed to counteract the methodological issues related to increasing SweTrau coverage and improved detection of NISS > 15 patients during the study period. Median values and quartiles were reported for variables with non-Gaussian distribution. The Python programming language was used for data management and analysis. A significance level of 0.05 was used for all analyses.

## Results

**Fig. 1:**
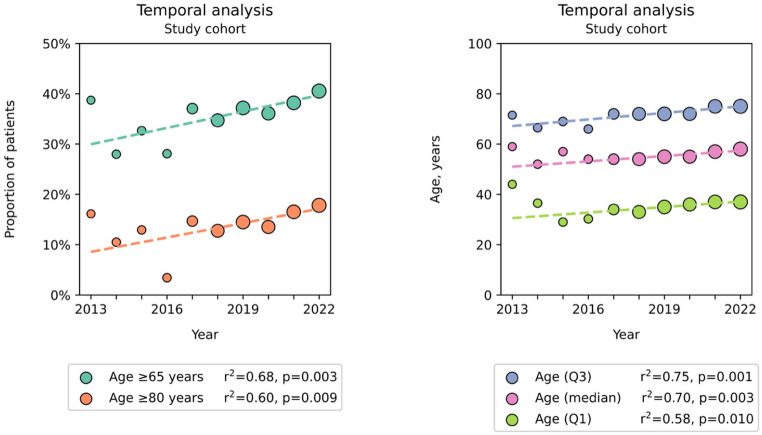
Temporal trends in the proportion of geriatric trauma patients and median age during the study period

11,969 patients were included in the study, of whom 4,451 were ≥ 65 years old, constituting 37% of all severely injured adult trauma patients during 2013–2022. The corresponding overall proportion of patients ≥ 80 years old was 15% (Table 1). There was an increase in the proportion of geriatric trauma patients during the study period, from approximately 30% in 2013 to 40% in 2022 according to the calculated values of the linear regression equation (R^2^ = 0.68, *p* = 0.003) (Fig. 1). The corresponding proportion of trauma patients ≥ 80 years old increased from approximately 8.6% to 17% during the study period (R^2^ = 0.60, *p* = 0.009) (Fig. 1). The median patient age also increased during the study period, from a weighted median of 51 years in 2013 to 57 years in 2022 (R^2^ = 0.70, *p* = 0.003) (Fig. 1).

**Table 1:** Baseline characteristics of the study cohort

Demographics	Study cohort *n* = 11,969	
**Patient characteristics:**		
Sex, (male: female), % (n)	72:28	(8673:3296)
Age, median (Q1, Q3)	56	(35, 73)
Age ≥ 65 years old, % (n)	37%	(4451)
Age ≥ 80 years old, % (n)	15%	(1785)
**Injury severity and mortality**:		
ISS, median (Q1, Q3)	17	(14, 25)
NISS, median (Q1, Q3)	22	(17, 30)
30-day mortality, % (n)	17%	(1997)
**Injury type**,** % (n)**:		
Blunt	92%	(10940)
Penetrating	8.4%	(1004)
**Injury intention**,** % (n)**:		
Accident	85%	(10018)
Violent assault	8.4%	(986)
Self-inflicted	6.0%	(699)
**Mechanism of injury**,** % (n)**:		
High fall	26%	(3068)
Low fall	19%	(2222)
Motor vehicle accident	15%	(1775)
Motorcycle accident	9.8%	(1164)
Bicycle accident	8.9%	(1058)
Hit/struck by blunt object	6.4%	(764)
Injured by knife/sharp object	5.5%	(655)
Injured pedestrian	3.4%	(403)
Gun shot wound	2.7%	(315)
Other vehicle	1.3%	(152)
Explosion injury	0.29%	(34)

**Fig. 2:**
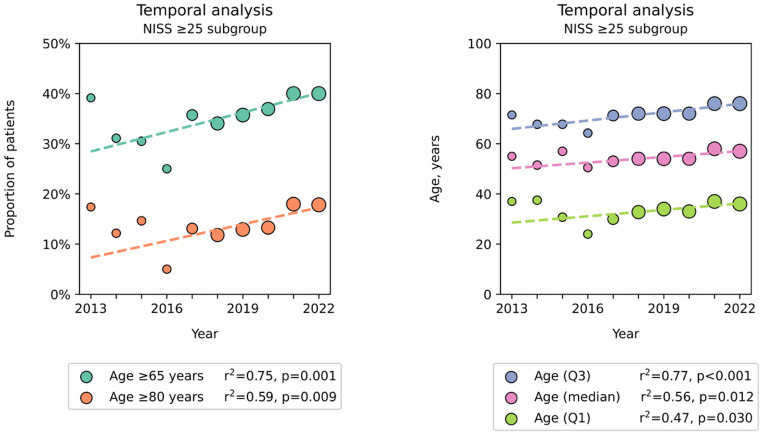
Temporal trend of geriatric trauma prevalence and median age in the NISS ≥ 25 subgroup

In the NISS ≥ 25 subgroup there was also a significant increase in the proportion of geriatric patients, with the proportion of patients ≥ 65 years old increasing from 28% to 40% (R^2^ = 0.75, *p* = 0.001) and patients ≥ 80 years old increasing from 7.3% to 17% (R^2^ = 0.59, *p* = 0.009) during the study period (Fig. 2). The median age increased from 50 to 57 years in this subgroup (R^2^ = 0.56, *p* = 0.012) (Fig. 2).

**Fig. 3:**
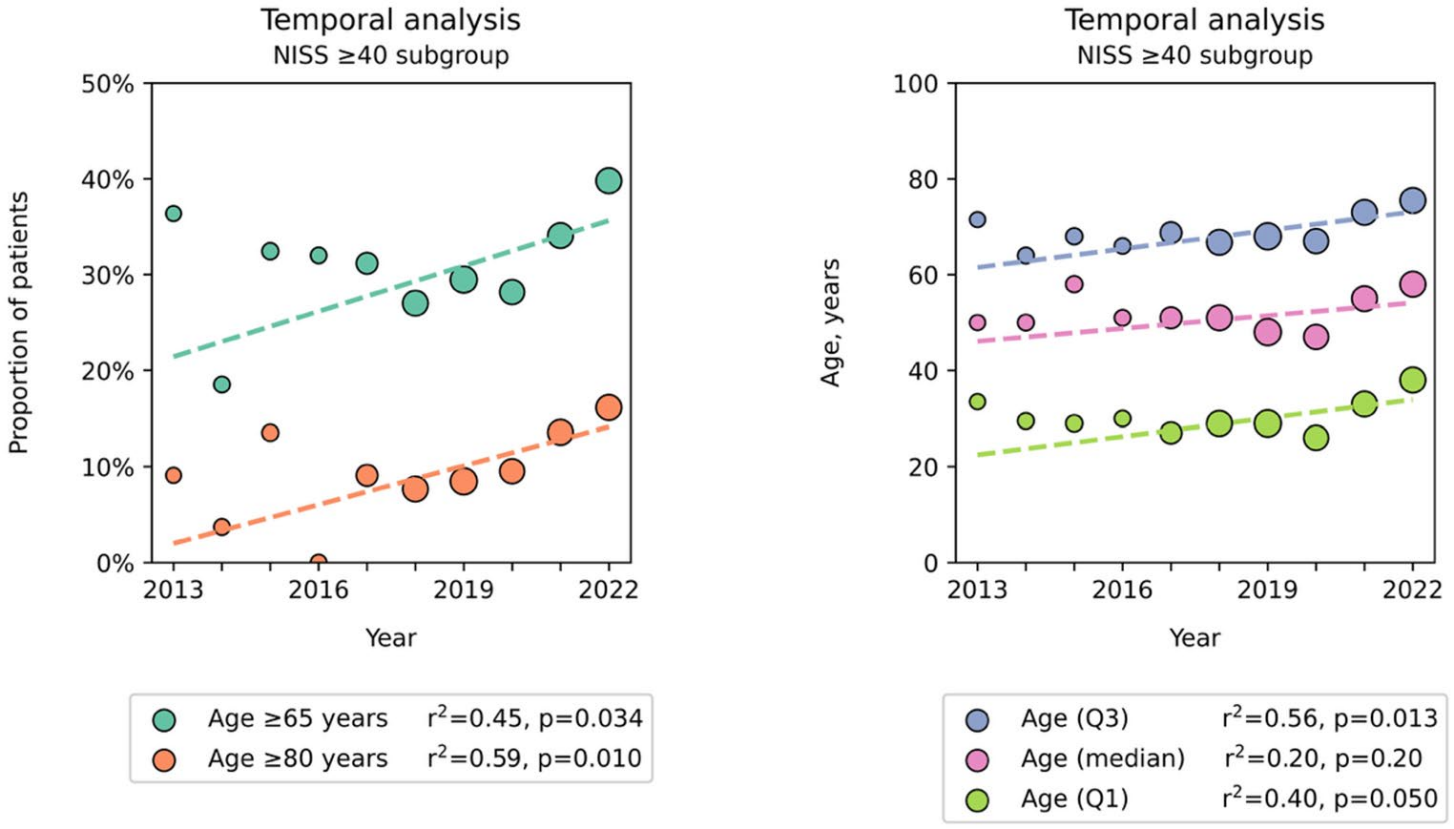
Temporal trend of geriatric trauma prevalence and median age in the NISS ≥ 40 subgroup

Finally, in the NISS ≥ 40 subgroup the overall proportion of patients ≥ 65 and ≥ 80 years old was 31% (*n* = 520) and 11% (*n* = 175) respectively (Table 2). There was a significant increase in the proportion of patients ≥ 65 years from 21% to 36% (R^2^ = 0.33, *p* = 0.085) and patients ≥ 80 years from 2.0% to 14% (R^2^ = 0.59, *p* = 0.010), with a corresponding increase in the median age in this subgroup from 46 to 54 years during the study period (R^2^ = 0.20, *p* = 0.20) (Fig. 3).

**Table 2:** Proportion of geriatric patients subdivided by injury severity

Demographics	Study cohort *n* = 11,969		NISS ≥ 25 *n* = 5369		NISS ≥ 40 *n* = 1651	
Age ≥ 65 years old, % (n)	37%	(4451)	37%	(1986)	31%	(520)
Age ≥ 80 years old, % (n)	15%	(1785)	15%	(785)	11%	(175)
Age, median (Q1, Q3)	56	(35, 73)	55	(34, 73)	52	(30, 70)

## Discussion

The present study aimed to determine the proportion of geriatric patients in the Swedish trauma population during 2013–2022 and analyze temporal trends in geriatric trauma prevalence. The overall proportion of geriatric patients in the adult trauma population was 37%, increasing from 30% to 40% during 2013–2022.

No previous studies have examined temporal trends geriatric trauma in Sweden. However, the proportion of elderly patients in the Swedish trauma population has been described in a study by Strömmer et al. which compared trauma patient mortality between university- and emergency hospitals during 2015–2018 [23]. The authors report geriatric patients constituted 31% of the trauma population, which is similar to the overall 37% found in the present study. However, there is likely significant overlap between the Strömmer cohort and the current study due to similar inclusion criteria and the use of SweTrau data in both studies [23]. These findings are also comparable to a Norwegian trauma epidemiology study by Cuevas-Østrem et al. examining patients from 2015 to 2018, where 33% of adult trauma patients were ≥ 65 years old [24]. Although Cuevas-Østrem et al. included patients with an NISS ≥ 9, this may still be an interesting comparison due to the similar demographic and socioeconomic factors between Sweden and Norway.

An earlier study by Sjögren and Björnstig examining geriatric trauma in Umeå, Sweden during 1985–1986 found that patients ≥ 60 years accounted for 15% of the total trauma population, while constituting 20% of inhabitants in Umeå [4]. By contrast, in 2022 the proportion of Swedish people ≥ 60 years old was 26% [1]. In other words, elderly people now constitute a greater proportion of the total population in Sweden today, which may be a contributing factor to the observed increasing prevalence of geriatric trauma patients seen in the present study. However, while this demographic shift may be a contributing factor, it is arguably not the sole explanation as the proportion of elderly trauma patients is growing faster than the corresponding share of elderly people in the general population. Nevertheless, it is likely that both factors play an important role in the shifting demographics of the modern trauma population in Sweden.

The results of the present study are in accordance with the demographic shift occurring in the Swedish population, where the average age is steadily rising [1, 2]. This trend is also seen in other affluent, developed nations with high HDI [17, 25]. Trauma has traditionally been considered to be the disease of young people, who predominantly sustain traumatic injuries through high-energy mechanisms of injury like motor vehicle accidents, violent assaults and falls from heights [26]. As the Swedish population ages and elderly patients constitute a larger share of the population, combined with an increased activity level among elderly people, a corresponding increase in the prevalence of geriatric is not unfeasible [3, 12, 26]. Sjögren and Björnstig also found that elderly patients accounted for 70% of the total costs of medical care in the seriously injured cohort, defined as the most severe injury scoring ≥ 3 according to the Abbreviated Injury Score (AIS) [4, 27].

These findings of high geriatric trauma prevalence may have several implications for the continued development of trauma care in Sweden. Previous studies have highlighted that geriatric patients may be neglected in modern trauma systems, and that current triage protocols may be limited in their ability to detect severe injuries in this population [11, 12, 14]. The validation study for the new Swedish TTA criteria also found that the new TTA criteria had worse performance in detecting severe injuries in elderly patients compared to their younger counterparts [28]. Another study by Lapidus et al., examining undertriage at one major non-trauma-center hospital in Stockholm, found that the overall undertriage rate of severely injured geriatric patients was 99% [14]. Geriatric-specific TTA criteria have been found to improve sensitivity of triage protocols for elderly trauma patients [29]. Given the shifting demographics of the Swedish trauma population, studies of alternative triage protocols adapted to geriatric trauma patients are warranted.

### Limitations

The present study has methodological limitations that should be acknowledged. Several studies suggest the primary mechanisms of injury in trauma are evolving, with an increasing prevalence of severely injured patients following low-energy mechanisms of injury such as ground-level falls and other low fall injuries [10, 30]. However, whether the increase in geriatric trauma observed in the present study is proportional to the demographic shift in the Swedish population is not known. This would require calculating an annual incidence of geriatric trauma adjusted for the geriatric population in Sweden at that time, with comparisons across the different age groups. Such analyses would require consistent national coverage of the SweTrau over several years. However, this data is currently not available. The lack of population-adjusted analyses is an acknowledged limitation of the present study, which should be considered in the interpretation of these results.

While geriatric trauma may be increasing, there are also alternative explanations. One such rather likely explanation may be an increasing coverage of SweTrau during the study period and improved data completeness in the registry [22]. Prior to 2022, not all Swedish emergency hospitals were reporting to SweTrau, and at the start of the study period in 2013 only about half of the hospitals in Sweden were connected to the registry [31]. Furthermore, a large proportion of those reporting data to the registry in the early days were university hospitals, which may have significantly different trauma patient demographics compared to regular emergency hospitals, such as a lower proportion of geriatric patients. Increasing national coverage during the study period and subsequent inclusion of new hospitals with higher proportion of elderly patients, could possibly explain the observed trend of increasing geriatric trauma. However, whether this trend is merely the result of time-varying ascertainment bias, the proportion of geriatric trauma patients during the latter years may still reflect the demographics of the Swedish trauma cohort, as SweTrau coverage has only ever increased over time. Consequently, while the exact rate at which the geriatric trauma population is increasing may be uncertain, the present study arguably disproves the notion that trauma is a disease of the young, as patients ≥ 65 years old constitute 40% of the severely injured adult trauma population in Sweden.

## Conclusion

There is likely an increase in geriatric trauma prevalence in Sweden, but the full extent of this temporal trend remains uncertain. Trauma patients ≥ 65 years old constitute 40% of severely injured adult trauma patients in Sweden. Further investigations of geriatric trauma prevalence in relation to demographic changes may be of interest to determine the future implications for Swedish healthcare.

## Data Availability

The data will not be published but is available via the corresponding author.

## Abbreviations

HDI: Human Development Index
NISS: New Injury Severity Score
TTA: Trauma team activation
